# Supplementary material for: Overt Word Reading and Visual Object Naming in Adults with Dyslexia: Electroencephalography Study in Transparent Orthography
Source: Bioengineering (Basel). 2024 May 4;11(5):459. doi: 10.3390/bioengineering11050459 (PMC11117949; doi:10.3390/bioengineering11050459)
Supplement: Supplementary file 1 [file bioengineering-11-00459-s001.zip › Figure S1.pdf]

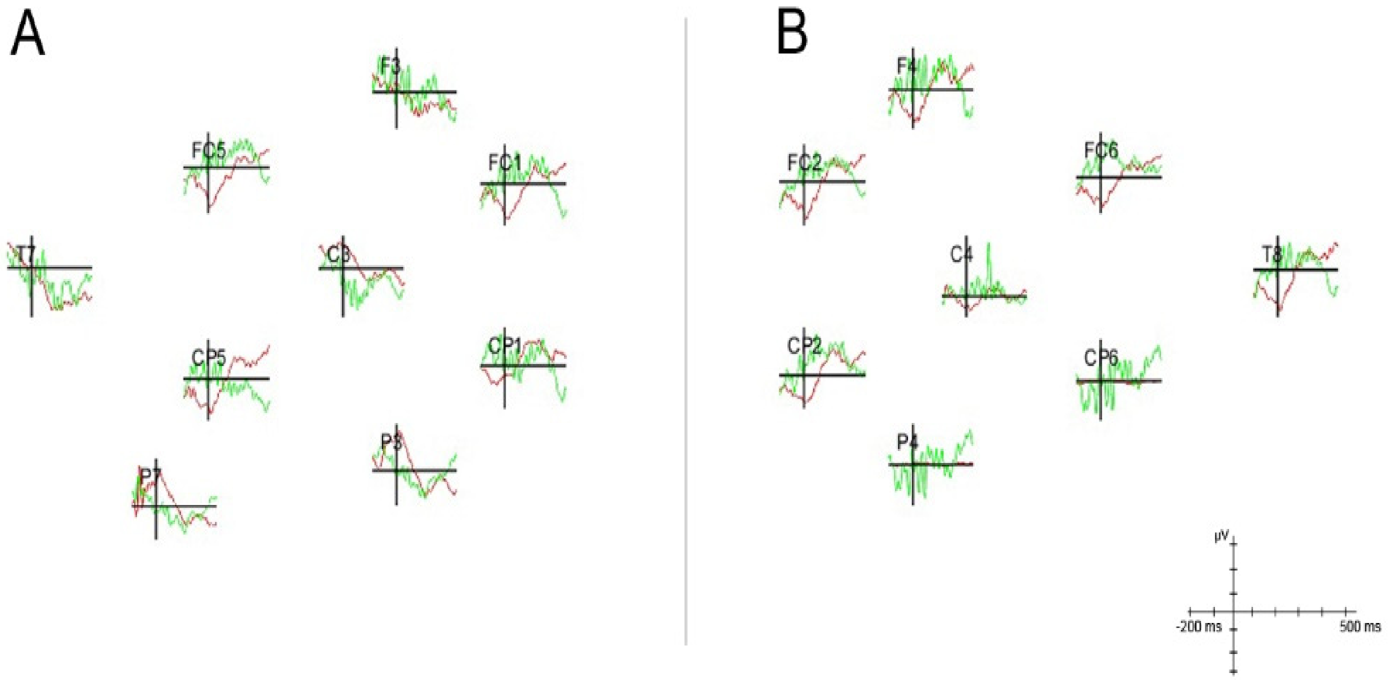

Figure S1. Grand average ERPs from ROI electrodes in overt reading task. A) Grand average ERPs from left ROI electrodes, B) Grand average ERPs from right ROI electrodes in overt reading task averaged separately for control participants (red line) and dyslexic participants (green line).
