# Supplementary material for: Overt Word Reading and Visual Object Naming in Adults with Dyslexia: Electroencephalography Study in Transparent Orthography
Source: Bioengineering (Basel). 2024 May 4;11(5):459. doi: 10.3390/bioengineering11050459 (PMC11117949; doi:10.3390/bioengineering11050459)
Supplement: Supplementary file 1 [file bioengineering-11-00459-s001.zip › Figure S2.pdf]

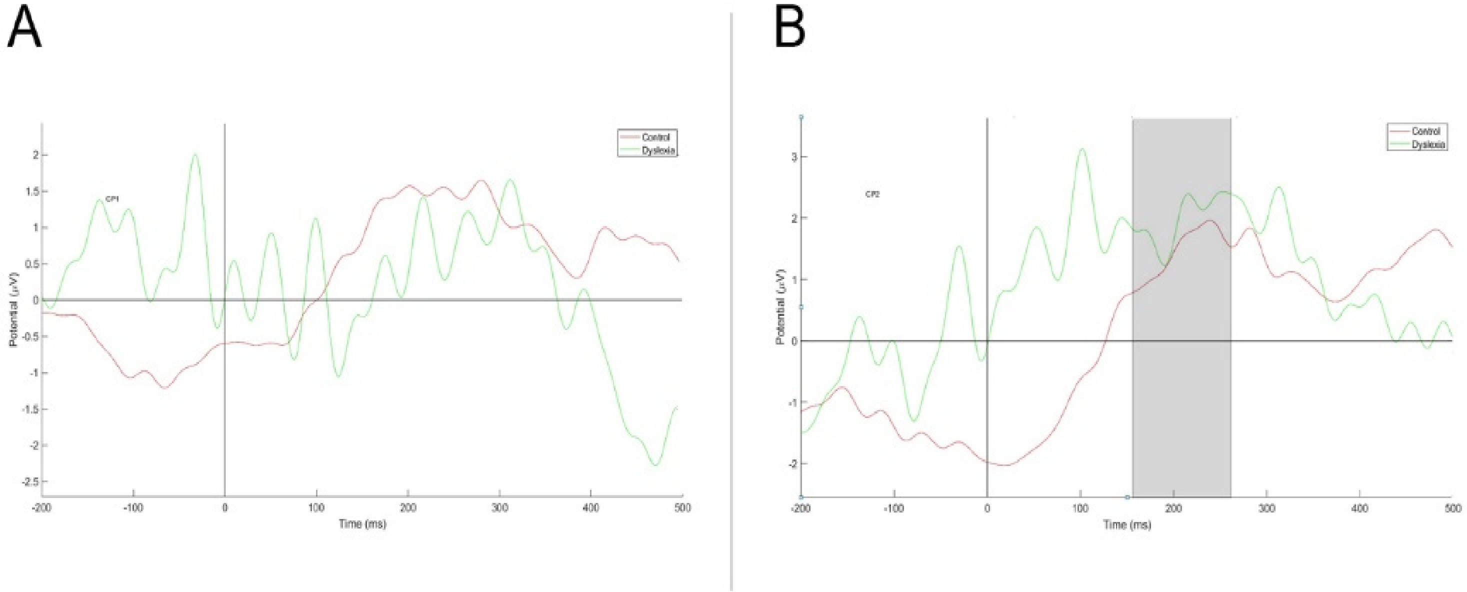

Figure S2. Schematic view of two ROI electrodes in overt reading task. A) CP1 electrode (left hemisphere), B) CP2 electrode (right hemisphere) showing a significant effect at 160-260 ms after picture presentation (gray area) at electrode CP2 in adult PDs compared to HCs in pre-lexical reading window (160-260 ms)
