# Supplementary material for: Overt Word Reading and Visual Object Naming in Adults with Dyslexia: Electroencephalography Study in Transparent Orthography
Source: Bioengineering (Basel). 2024 May 4;11(5):459. doi: 10.3390/bioengineering11050459 (PMC11117949; doi:10.3390/bioengineering11050459)
Supplement: Supplementary file 1 [file bioengineering-11-00459-s001.zip › Figure S3.pdf]

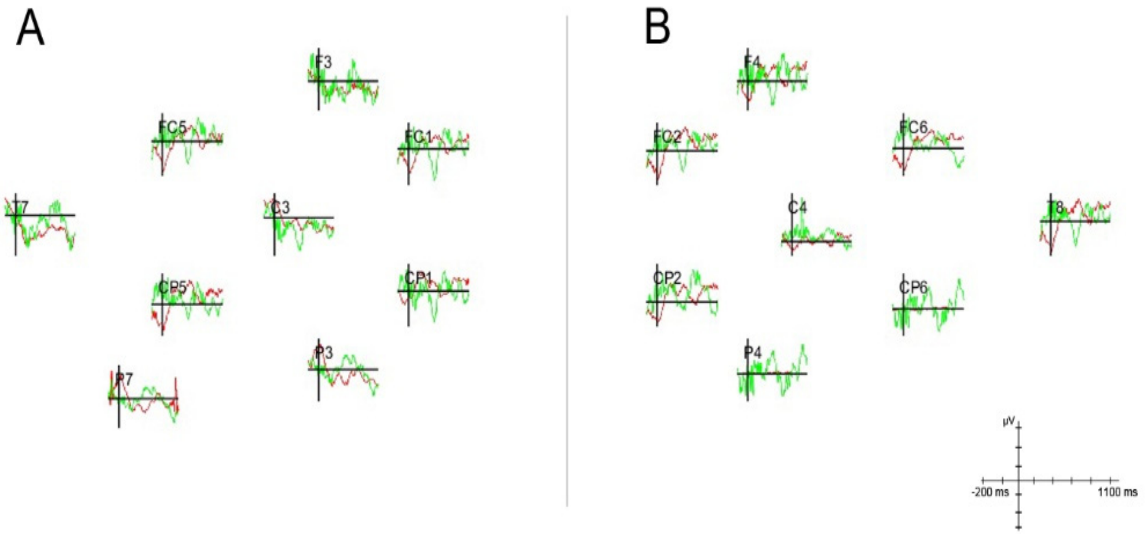

Figure S3. Grand average ERPs from ROI electrodes in overt object naming task. **A)** Grand average ERPs from left ROI electrodes, **B)** Grand average ERPs from right ROI electrodes in overt object naming task averaged separately for control participants (red line) and dyslexic participants (green line).
