# Supplementary material for: Overt Word Reading and Visual Object Naming in Adults with Dyslexia: Electroencephalography Study in Transparent Orthography
Source: Bioengineering (Basel). 2024 May 4;11(5):459. doi: 10.3390/bioengineering11050459 (PMC11117949; doi:10.3390/bioengineering11050459)
Supplement: Supplementary file 1 [file bioengineering-11-00459-s001.zip › Figure S4.pdf]

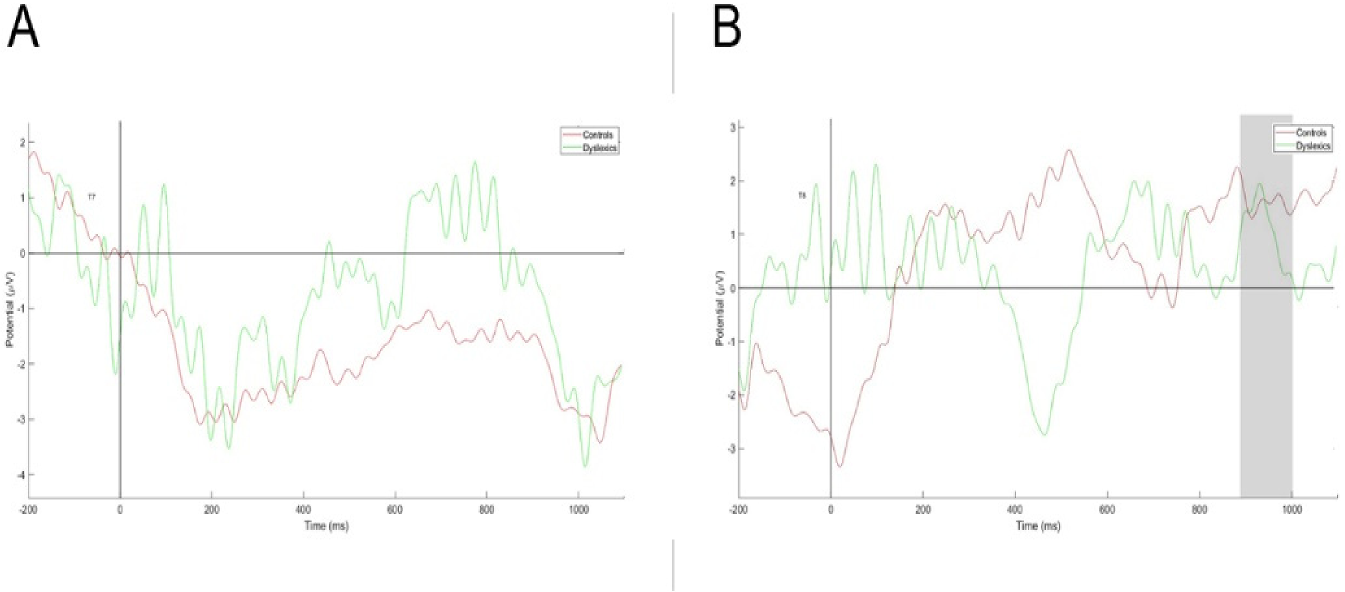

Figure S4. Schematic view of two ROI electrodes in overt object naming task. A) T7 electrode (left hemisphere), B) T8 electrode (right hemisphere) showing a significant effect at 900-1000 ms after picture presentation (grey area) at electrode T8 in adult PDs compared to HCs in post-lexical naming window (900-1000ms)
