# Supplementary material for: Overt Word Reading and Visual Object Naming in Adults with Dyslexia: Electroencephalography Study in Transparent Orthography
Source: Bioengineering (Basel). 2024 May 4;11(5):459. doi: 10.3390/bioengineering11050459 (PMC11117949; doi:10.3390/bioengineering11050459)
Supplement: Supplementary file 1 [file bioengineering-11-00459-s001.zip › Table S1.pdf]

Supplementary material

Table S1. Naming blocks (picture stimuli)

1. Block

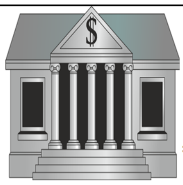 *	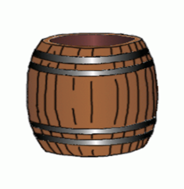	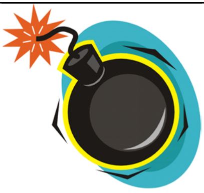	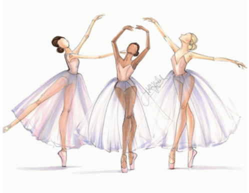 *
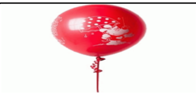	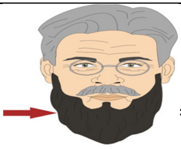 *	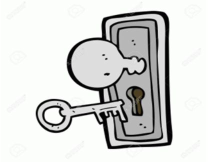	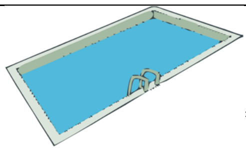 *
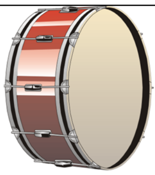	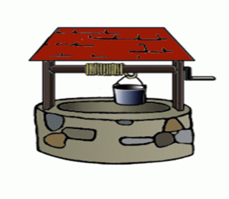	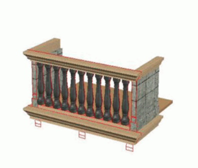 *	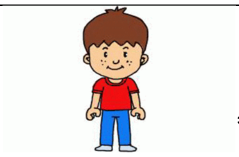 *
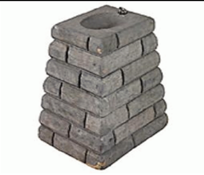	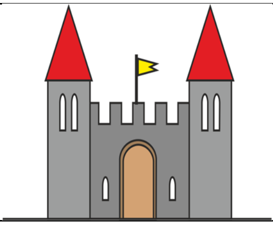	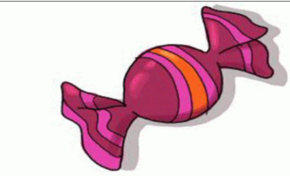	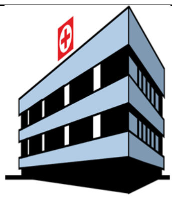 *

2.. Block

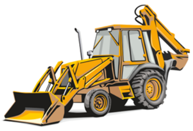	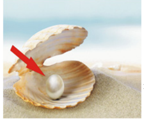 *	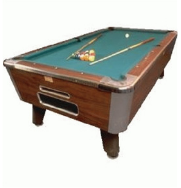	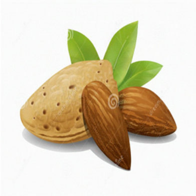
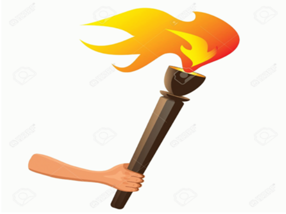	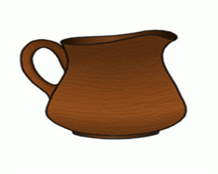	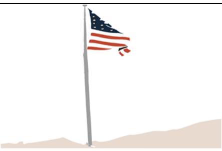 *	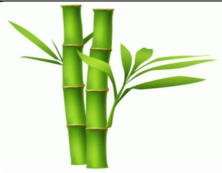

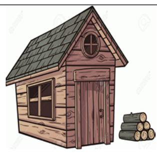	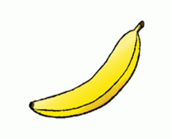	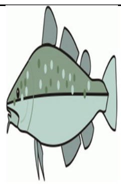	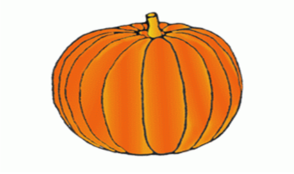
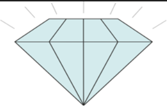	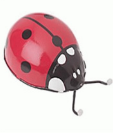 *	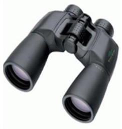	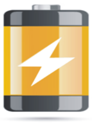

3.Block

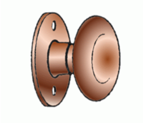	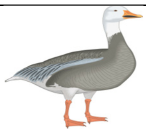	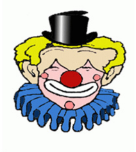	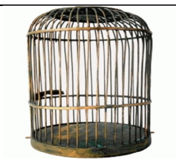 *
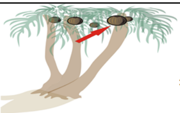 *	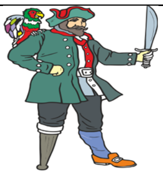	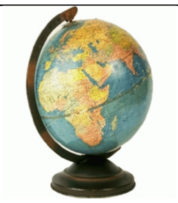	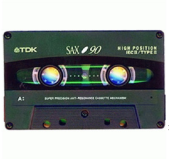 *
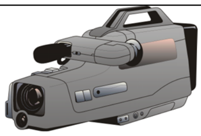	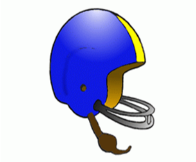 *	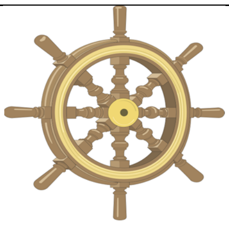	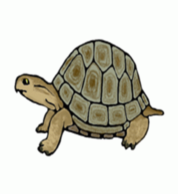
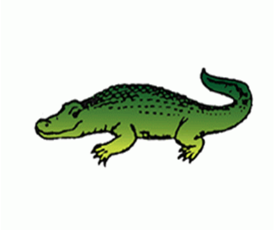	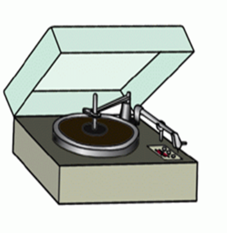	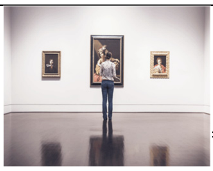 *	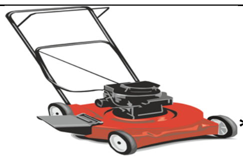 *

4. Block

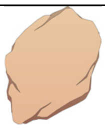	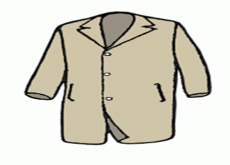	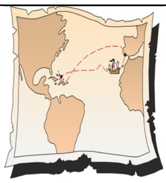	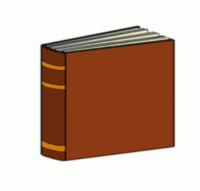
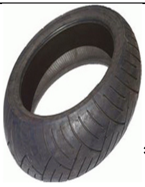 *	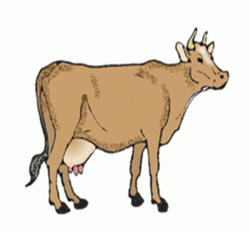	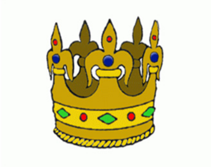	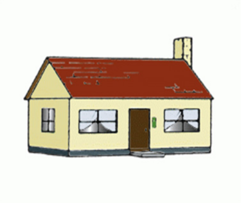
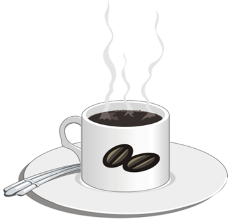	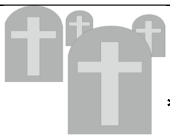 *	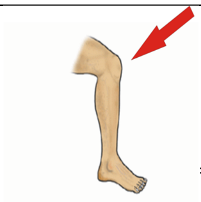 *	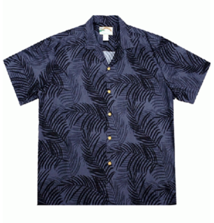
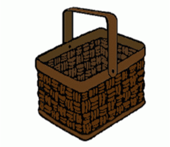	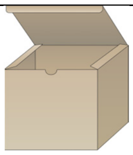	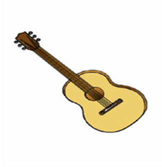	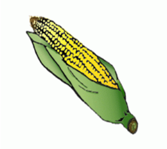

Fourty five picture stumuli were choosen from Revisiting Snodgrass and Vanderwart's pictorial object set [108] and nineteen* picture stimuli from Corel Gallery™Magic [109]. Pictures shared same names as words provided in Suplementary Table 2.

** Pictures stimuli from Corel Gallery™Magic [109]*
