# Supplementary material for: Overt Word Reading and Visual Object Naming in Adults with Dyslexia: Electroencephalography Study in Transparent Orthography
Source: Bioengineering (Basel). 2024 May 4;11(5):459. doi: 10.3390/bioengineering11050459 (PMC11117949; doi:10.3390/bioengineering11050459)
Supplement: Supplementary file 1 [file bioengineering-11-00459-s001.zip › Table S2.pdf]

Supplementary material

Table S2. Reading blocks stimuli

1. **Block reading words**

	**Word (Croatian)**	***Translated to English***	**Letter number**	**Syllable number**	**Apsolute frequency**	**Relative Frequency**
1.	bager	*excavator*	5	2	569	0,0001
2.	biser	*pearl*	5	2	569	0,0001
3.	biljar	*billiards*	5	2	569	0,0001
4.	badem	*almond*	5	2	568	0,0002
5.	baklja	*torch*	5	2	562	0,0008
6.	bokal	*carafe*	5	2	567	0,0003
7.	barjak	*banner*	6	2	553	0,0017
8.	bambus	*bamboo*	6	2	568	0,0002
9.	baraka	*shack*	6	3	562	0,0008
10.	banana	*banana*	6	3	565	0,0005
11.	bakalar	*cod*	7	3	567	0,0003
12.	bundeva	*pumpkin*	7	3	567	0,0003
13.	dijamant	*diamond*	8	3	560	0,0010
14.	bubamara	*ladybug*	8	4	567	0,0003
15.	dalekozor	*binoculars*	8	4	562	0,0008
16.	baterija	*battery*	8	4	566	0,0004

2. Block reading words

	Word (Croatian)	*Translated to English*	Letter number	Syllable number	Apsolute frequency	Relative Frequency
1.	banka	*bank*	5	2	444	0,0126
2.	bačva	*barrel*	5	2	538	0,0032
3.	bomba	*bomb*	5	2	493	0,0077
4.	balet	*ballet*	5	2	547	0,0023
5.	balon	*balloon*	5	2	559	0,0011
6.	brada	*beard*	5	2	511	0,0059
7.	brava	*lock*	5	2	549	0,0021
8.	bazen	*pool*	5	2	550	0,0020
9.	bubanj	*drum*	5	2	535	0,0035
10	bunar	*well*	5	2	530	0,0040
11.	balkon	*balcony*	6	2	541	0,0029
12.	dječak	*boy*	6	2	415	0,0155
13.	dimnjak	*chimney*	6	2	537	0,0033
14.	dvorac	*castle*	6	2	553	0,0017
15.	bombon	*candy*	6	2	560	0,0010
16.	bolnica	*hospital*	7	3	462	0,0108

3. **Block of words**

	Word (Croatian)	*Translated to English*	Letter number	Syllable number	Apsolute frequency	Relative Frequency
1.	kvaka	*doorknob*	5	2	556	0,0014
2.	guska	*goose*	5	2	560	0,0010
3.	klaun	*clown*	5	2	568	0,0002
4.	kavez	*cage*	5	2	558	0,0012
5.	kokos	*coconut*	5	2	568	0,0002
6.	gusar	*pirate*	5	2	566	0,0004
7.	globus	*globe*	6	2	558	0,0012
8.	kaseta	*tape*	6	3	569	0,0001
9.	kamera	*camera*	6	3	568	0,0002
10.	kaciga	*helmet*	6	3	559	0,0011
11.	kormilo	*rudder*	7	3	565	0,0005
12.	kornjača	*turtle*	7	3	559	0,0011
13.	krokodil	*crocodile*	8	3	557	0,0013
14.	gramofon	*record player*	8	3	559	0,0011
15.	galerija	*gallery*	8	4	551	0,0019
16.	kosilica	*lawnmower*	8	4	569	0,0001

4. **Block of words**

	**Word (Croatian)**	*Translated to English*	**Letter number**	**Syllable number**	**Apsolute frequency**	**Relative Frequency**
1.	Kamen	*stone*	5	2	284	0,0295
2.	Kaput	*coat*	5	2	501	0,0069
3.	Karta	*map*	5	2	453	0,0117
4.	Knjiga	*book*	5	2	323	0,0248
5.	Guma	*tire*	5	2	535	0,0035
6.	Krava	*cow*	5	2	477	0,0093
7.	Kruna	*crown*	5	2	518	0,0052
8.	Kuća	*house*	5	2	99	0,0937
9.	Kava	*coffee*	5	2	512	0,0058
10.	Groblje	*cemetery*	5	2	501	0,0069
11.	Koljeno	*knee*	6	3	449	0,0021
12.	Košulja	*shirt*	6	3	470	0,0100
13.	Kutija	*box*	6	3	479	0,0091
14.	Gitara	*guitar*	6	3	547	0,0023
15.	Košara	*basket*	6	3	543	0,0027
16.	Kukuruz	*corn*	7	3	521	0,0049
