# Supplementary material for: Overt Word Reading and Visual Object Naming in Adults with Dyslexia: Electroencephalography Study in Transparent Orthography
Source: Bioengineering (Basel). 2024 May 4;11(5):459. doi: 10.3390/bioengineering11050459 (PMC11117949; doi:10.3390/bioengineering11050459)
Supplement: Supplementary file 1 [file bioengineering-11-00459-s001.zip › Table S3.pdf]

Supplementary material

**Table S3.** Descriptive means and standard deviations based on observation of amplitudes (μV) for different time windows.

| Time window | Group | Hemisphere | Word reading M | Word reading sd | Visual object naming M | Visual object naming sd |
|---|---|---|---|---|---|---|
| Pre-lexical | Control | Right | 0.37 | 3.28 | 0.64 | 2.89 |
| | | Left | -0.43 | 3.68 | -0.64 | 3.90 |
| | Dyslexia | Right | 0.29 | 6.95 | 1.17 | 5.61 |
| | | Left | -0.71 | 3.81 | -0.76 | 4.50 |
| Lexical | Control | Right | 1.08 | 2.54 | 0.78 | 3.44 |
| | | Left | -0.36 | 2.96 | -0.38 | 3.90 |
| | Dyslexia | Right | 0.36 | 4.96 | 0.08 | 6.64 |
| | | Left | 0.25 | 4.19 | -0.63 | 4.17 |
| Post-lexical | Control | Right | 1.17 | 3.21 | 1.07 | 2.20 |
| | | Left | -0.44 | 3.93 | -0.32 | 2.81 |
| | Dyslexia | Right | 0.73 | 3.53 | 0.08 | 3.98 |
| | | Left | -0.19 | 2.69 | -0.69 | 2.68 |
